# Factors Influencing Pressure Injury Development and Survival Duration in Adults Admitted to the ICU: A Retrospective Cohort Study Following the STROBE Guidelines

**DOI:** 10.3390/healthcare13121411

**Published:** 2025-06-12

**Authors:** Warantorn Potarin, Santisith Khiewkhern, Thidarat Somdee, Chitkamon Srichompoo, Kemika Sombateyotha, Jirarat Ruetrakul, Le Ke Nghiep, Kukiat Tudpor

**Affiliations:** 1 Faculty of Nursing, Srimahasarakham Nursing College, Praboromarajchanok, Maha Sarakham 44000, Thailand; 63011490007@msu.ac.th; 2 Faculty of Public Health, Mahasarakham University, Maha Sarakham 44150, Thailand; thidarat@msu.ac.th (T.S.); kemika.s@msu.ac.th (K.S.); kukiat.t@msu.ac.th (K.T.); 3 Public Health and Environmental Policy in Southeast Asia Research Cluster (PHEP-SEA), Mahasarakham University, Mahasarakham 44150, Thailand; 4 Faculty of Health and Sports Science, Thaksin University Phatthalung Campus, Phatthalung 93210, Thailand; chitkamon.s@msu.ac.th; 5 Faculty of Nursing, Naresuan University, Phitsanulok 65000, Thailand; ajira2512@gmail.com; 6 Vinh Long Department of Health, Vinh Long 85000, Vietnam; lekenghiep@gmail.com

**Keywords:** pressure ulcer, bed sores, survival duration, hospitalized patients, intensive care unit

## Abstract

**Background:** Individuals receiving intensive care are particularly vulnerable to developing pressure sores. This heightened risk necessitates a thorough understanding of relevant risk factors and the time at which these injuries manifest, facilitating effective prevention. **Objective:** This investigation, structured as a retrospective cohort study, aimed to assess the duration until pressure sore onset and identify contributing risk factors among 50 adult patients in an intensive care unit (ICU), observed over 12 weeks. **Methodology:** Our analysis employed the Kaplan–Meier approach for survival analysis. We then used the log-rank test to determine significant differences in survival curves. We utilized COX proportional regression analysis to explore the associations between various factors and the development of pressure injuries during the ICU stay. **Results:** Data from 50 adult ICU patients showed that 29 patients developed pressure sores. Coccyx (44%) and back (6%) were most affected. The median survival time from ICU admission to the final skin examination for pressure sore development was 3 days. The multivariable Cox regression analysis revealed that males with a high BMI, increased systolic blood pressure, elevated albumin levels, and a more extended ICU stay were at a significantly higher risk of developing pressure ulcers (*p*-value < 0.05). **Conclusions:** The research highlights the need to prioritize males with high BMI, high blood pressure, and high albumin patients in preventing pressure sores in the ICU, with an extended ICU stay significantly increasing the risk. This information can be utilized to develop clinical guidelines for reducing pressure sore incidence and improving patient care.

## 1. Introduction

Pressure injuries—also known as pressure ulcers or pressure sores—are areas of localized damage to the skin and underlying tissues, typically caused by prolonged pressure, shear, or friction, especially over bony prominences [1,2]. They are multifactorial in origin, influenced by limited mobility, poor nutrition, moisture imbalance, impaired circulation, and mechanical devices that apply sustained pressure to the skin [3,4]. Contrary to the belief that these injuries develop gradually, they can occur within 2 h of continuous pressure application [5]. The internationally recognized staging system classifies pressure injuries from Stage I to Stage IV, including unstageable lesions and suspected deep tissue injuries, based on the depth and severity of tissue damage [6].

Among hospitalized populations, patients in intensive care units (ICUs) face the highest risk of developing pressure injuries due to immobility, hemodynamic instability, reduced tissue perfusion, impaired oxygenation, and prolonged exposure to medical devices [7,8,9,10]. The increased longevity and complexity of care among critically ill and geriatric patients have also contributed to higher rates of pressure injuries in ICUs globally [11,12,13]. Within the nursing practice domain, pressure sores act as a significant benchmark reflecting the standard of care provided by nurses and the overall proficiency of healthcare practitioners. Nurses are instrumental in proactively averting these lesions and ensuring a swift intervention upon their appearance [14,15,16].

A growing body of literature has explored the epidemiology, risk factors, and outcomes of pressure injuries in ICU settings. Most existing studies have adopted retrospective designs or cross-sectional analyses, often focusing on the prevalence and incidence rates or evaluating isolated risk factors using logistic regression models [6,8,10]. While these studies have provided important insights, they often lack a comprehensive investigation into the cumulative effect of multiple interacting variables, such as survival duration, medical device exposure, and physiological instability, on pressure injury development in critically ill patients. Furthermore, few studies have incorporated time-dependent factors or survival analysis approaches to predict pressure injury risk throughout ICU stay.

This study aimed to address these gaps by investigating the relationship between survival duration and pressure injury development in ICU patients through a robust analytical framework. By integrating clinical and device-related risk factors using multivariate modeling and survival analysis, this research provides a more dynamic understanding of pressure injury risk over time. The findings inform proactive, individualized prevention strategies and enhance nursing care quality in high-risk populations. The study has been built upon existing literature by employing a longitudinal approach that captures the temporal dynamics of pressure injury development. The originality of this research lies in its methodological emphasis on survival duration as a predictive factor, its consideration of multiple coexisting risk factors, and its application within an ICU context. These elements provide a novel contribution to the field and directly support the study’s aim to identify and quantify the effects of survival duration and other risk factors on developing pressure injuries among ICU patients.

## 2. Materials and Methods

### 2.1. Study Design and Participants

This retrospective cohort study aimed to investigate the occurrence of pressure injuries among 595 critically ill patients admitted to the adult intensive care unit (ICU) at Mahasarakham Hospital, Thailand, between 1 January 2023 and 31 October 2024. Following the STROBE (Strengthening the Reporting of Observational Studies in Epidemiology) guidelines, the study was designed and reported to ensure methodological transparency and reproducibility. Data was collected on a single study day, capturing baseline clinical and demographic information. Patients were subsequently followed for 12 weeks to assess the development of pressure injuries, survival status, and length of hospital stay.

The sample size was calculated using G*Power version 3.1, assuming a large effect size of 0.25, an alpha level of 0.05, and a power of 0.95. The required sample size was 49 patients. Sample selection was conducted using simple random sampling by the lottery method from a list of patients who met the eligibility criteria.

Fifty eligible participants included adult patients aged 18 and older admitted to the ICU between 00:00 and 23:59:59 on the designated study day. Inclusion criteria required a minimum ICU stay of three days and the provision of informed consent by either the patient or a legally authorized representative. Patients were excluded if they had severe clinical conditions that precluded participation, if consent was declined, or if the patient died during the study period.

### 2.2. Data Collection

Data collection was conducted on 1 January 2023. Anonymous patient information was obtained using standardized case report forms, which included data extracted from electronic medical records from HOSxP (Hospital Information System) within the first three days of ICU admission. Collected variables encompassed demographic characteristics, admission details, and physiological parameters relevant to the study day. Disease severity was assessed using the Simplified Acute Physiology Score II (SAPS II) (AUC = 0.85) [17,18]. Pressure injuries were systematically recorded through direct clinical observation, following internationally recognized staging definitions [6]. Pressure injury risk was evaluated using the Braden Scale, which consists of six subscales—mobility, activity, sensory perception, skin moisture, nutritional status, and friction/shear—with lower scores indicating higher risk (AUC = 0.82) [19,20]. While the SAPS II and Braden Scale are widely used internationally, their use in this study included content validation by three experts for relevance and appropriateness in the Thai clinical context. However, formal cross-cultural validation or psychometric testing in the Thai population was not established and is acknowledged as a limitation.

This study’s staging of pressure ulcers followed the classification system established by the National Pressure Ulcer Advisory Panel (NPUAP), which categorizes pressure injuries into six stages, including device-related and mucosal membrane pressure injuries. Assessments were conducted collaboratively by experienced ICU nurses and attending physicians to ensure accuracy and consistency. Follow-up data were collected on survival status, length of ICU stay, and overall hospital stay, either until discharge or up to 12 weeks post-admission. To ensure consistency and accuracy in pressure injury assessment, a training module—accompanied by a self-assessment tool based on international staging definitions [6]—was developed and included in the official study guidelines. This module underwent content validation by three experts before the study’s commencement. All registered data collectors were instructed and encouraged to thoroughly review the training materials before participating in the data collection process.

### 2.3. Data Management

The reported data underwent a thorough evaluation for its quality and integrity. Any missing values that appeared extreme or seemed unlikely were cross-referenced directly with the patients’ medical records. If the data still raised concerns, the principal investigator made the final decision regarding its inclusion in the study, reaching a consensus with the team. Missing values deemed suitable for inclusion through this agreement were either filled in using median values or inferred from other existing variables. Any remaining missing values were then excluded from the final analyses.

### 2.4. Statistical Analysis

The statistical analyses were conducted at the individual patient level. To determine the proportion of pressure sores occurring in the ICU on the study day, we calculated the percentage of patients with at least one pressure injury. Continuous data were summarized using the median and interquartile range (IQR), while categorical data were presented as counts (n) and percentages (%). Univariate analyses were conducted using appropriate statistical tests, including the Chi-square test, Exact test, Mann–Whitney U test, and Kruskal–Wallis test. Survival analysis was conducted using the Kaplan–Meier procedure, and the log-rank test was used to assess differences in survival curves. COX proportional regression analysis was employed to examine associations with ICU-acquired pressure injuries. All variables were included in the analysis, regardless of their results in the univariate analysis. The results are reported as hazard ratios (HRs) with corresponding 95% confidence intervals (CIs). The statistical analysis was performed using IBM SPSS version 25.0, licensed by Mahasarakham University.

### 2.5. Ethical Considerations and Informed Consent

This research was approved by the Human Research Ethics Committee at Mahasarakham University, with accreditation number 419-383/2565. Before inclusion in the study, the relatives of patients in the sample were informed about the study’s objectives. They were asked to provide voluntary informed consent to participate, with the option to decline participation at any time. The collected data will be treated confidentially, ensuring that respondents remain anonymous, and the results will be presented in a manner that preserves their anonymity. All research procedures were conducted in compliance with relevant guidelines and regulations.

## 3. Results

### 3.1. Demographic Characteristics and Health Condition

The study’s participant pool was predominantly male, accounting for 76.00% of the cohort, with an average age of 56.74 ± 11.53 years. Their mean body mass index (BMI) was 22.16 ± 4.53 kg/m^2^. A key characteristic was that every participant (100.00%) required mechanical ventilation and received complete medical and nursing care. Clinical observations showed an average temperature of 37.89 ± 0.94 °C. Blood pressure readings indicated a mean systolic pressure of 126.00 ± 27.91 mmHg and a mean diastolic pressure of 72.70 ± 16.39 mmHg. The mean arterial pressure (MAP) averaged 87.26 ± 16.10 mmHg, with a remarkable 98.00% of individuals falling within the typical normal range of 65.00–110.00 mmHg. Beyond physiological measures, the participants generally exhibited good health habits and functional status. Most reported good sleep quality (60.00%), and all were non-smokers (100.00%). Regular bowel movements, specifically once per day, were reported by 76.00% of participants. A significant majority received standard inotropic support (58.0%), displayed normal respiratory mechanics (86.0%), and maintained complete independence in their activities of daily living (ADLs) (100.0%). Regarding pre-existing health conditions, diabetes mellitus (DM) was the most common, affecting 30.00% of participants, followed by hypertension (HT) at 24.00%. Importantly, no cases of edema, urinary incontinence, additional comorbidities, or any alterations in the level of consciousness (LOC) were found in this study group.

An assessment of pressure sore classification using the Barden score indicated that most were grade 2 (28.00%), followed by grade 1 (22.00%) and grade 3 (8.00%). The average duration of ICU admission was 7.60 ± 8.03 days. Most participants (74.00%) had an ICU stay of less than 9 days. The average time for a pressure sore to develop was 3.66 ± 4.90 days. Furthermore, most participants presented with abnormal albumin levels (92.00%), either above 5.00 gm/dL or below 3.50 gm/dL. Abnormal hemoglobin levels (below 11.00 g/dL) were also noted in 58.00% of cases. Detailed information is presented in Table 1.

### 3.2. Location of Pressure Sore

**Figure 1:**
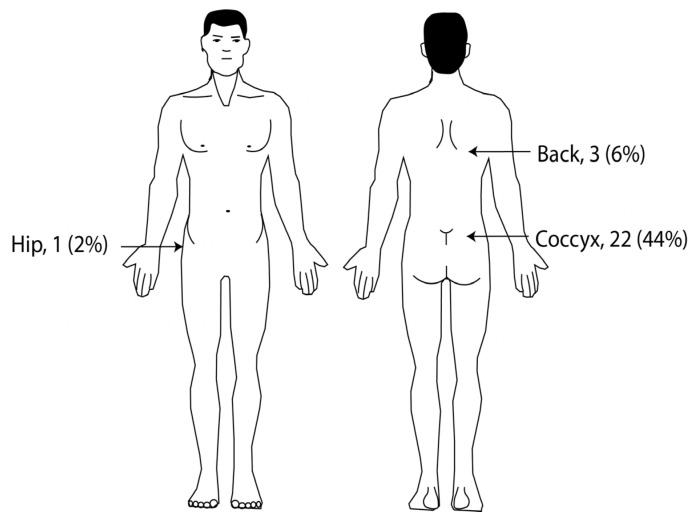
Anatomical locations of pressure sores in ICU patients (n = 50).

An analysis of pressure sore locations showed the coccyx was most frequently affected, appearing in 44% of patients. Less common sites for these lesions included the back (6%) and the hip (2%), as detailed in Figure 1.

### 3.3. Survival Time of Pressure Sore Development

**Figure 2:**
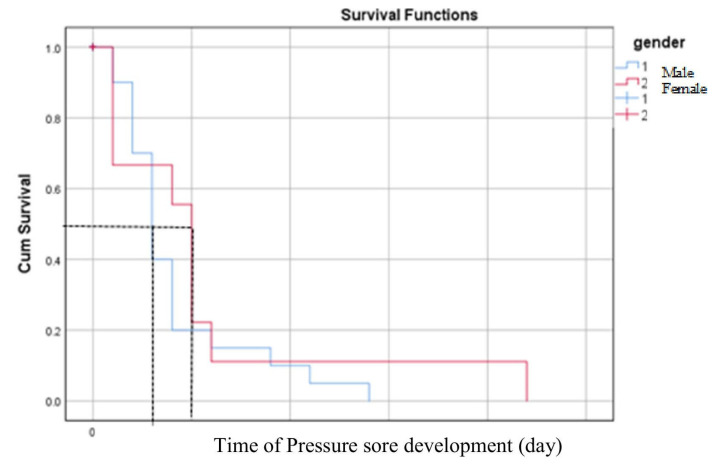
Medians for survival time of pressure sore development comparison between male and female. The black dashed lines indicate the median day of survival time for pressure sore development.

The comprehensive survival analysis for pressure sore development in ICU patients indicated a median survival time of 3 days (95% CI = 2.04 to 3.96). While males exhibited a shorter median time to pressure sore development at 3 days (95% CI = 2.28 to 3.72), compared to females at 5 days (95% CI = 4.18 to 5.82), this difference was not statistically significant (*p*-value = 0.389) at the α ≤ 0.05 level, as illustrated in Table 2 and Figure 2.

### 3.4. Comparative Analysis of Survival Time for Pressure Sore Development at the Mean of Covariates

**Figure 3:**
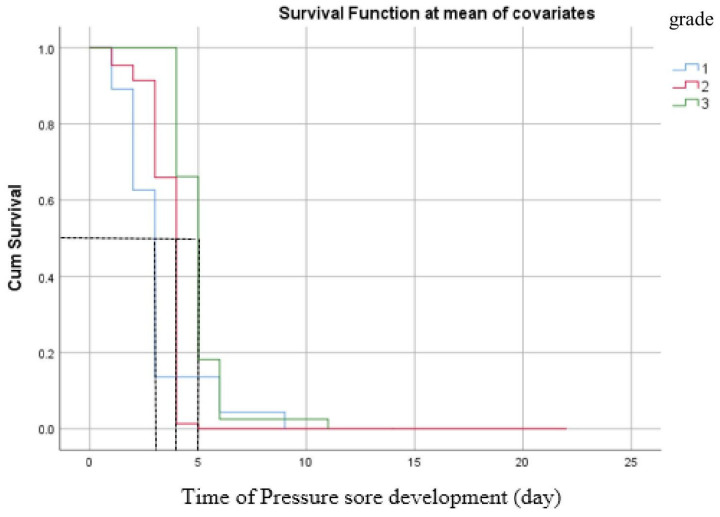
Survival time of pressure sore development at the mean of covariates. The black dashed lines indicate the median day of survival time for pressure sore development.

The results showed that pressure sore stage 3 has a longer median survival time of 5 days, stage 2 exhibited a median survival time of 4 days, while stage 1 had a shorter median survival time (3 days, 95% CI, 1.49 to 4.51 days). However, this difference is not statistically significant at a *p*-value ≤ 0.05, as shown in Figure 3.

### 3.5. Multivariable Analysis of Risk Factors for Pressure Sores Development

The study employed multivariable Cox proportional regression with the Backward method to assess the risk factors associated with pressure sore development in adult ICU patients. Several of the fourteen factors analyzed differed significantly between the two groups, with *p*-values ≤ 0.25. These factors included age (years), gender, body mass index (BMI, kg/m^2^), temperature (°C), systolic blood pressure (mmHg), diastolic blood pressure (mmHg), mean arterial pressure (MAP, mmHg), presence of diabetes mellitus (DM), hypertension (HT), serum albumin (g/dL), hemoglobin level (g/dL), and length of ICU stay (<9 days vs. ≥9 days).

The results indicated that males exhibited a statistically significant hazard ratio for pressure sore development, which was four times higher (95% CI = 1.25 to 12.10) than that of females. BMI also demonstrated a statistically significant hazard ratio for pressure sore development, with a 1 kg/m^2^ increase associated with a 0.22 times higher hazard ratio at a *p*-value of 0.001. Systolic blood pressure showed a statistically significant hazard ratio for pressure sore development; a 1 mmHg increase was associated with a 0.04 times lower hazard ratio at a *p*-value of 0.003. Albumin displayed a statistically significant hazard ratio for pressure sore development, with a 1 gm/dL increase associated with a 1.15 times higher hazard ratio at a *p*-value of 0.001. Moreover, a length of stay in the ICU of 9 days or more exhibited a statistically significant hazard ratio for pressure sore development, which was 8.27 times higher (95% CI = 2.42 to 28.28) compared to those with a length of stay in the ICU of less than 9 days (*p*-value = 0.001). However, age, diastolic blood pressure, mean arterial pressure (MAP), diabetes (DM), hypertension (HT), and hemoglobin were not statistically significant for pressure sore development in ICU patients, as presented in Table 3.

## 4. Discussion

This retrospective cohort study evaluated the time to onset of pressure sores and associated risk factors in a cohort of 50 adult ICU patients over a 12-week interval. We found that most ICU patients with pressure ulcers were older, which was not statistically significant compared to prior studies, potentially due to varied patient populations and care. Secondly, incomplete data on comorbidities limited the study, but being male significantly increased pressure sore risk, and surprisingly, higher BMI showed a slight increase in risk, opposing previous findings. Thirdly, longer ICU stays and albumin were significant risk factors for pressure sores, though diabetes and hypertension did not show a direct, independent effect on development time. Fourthly, despite prior research linking prolonged mechanical ventilation to pressure sores, this study, where all patients were ventilated, found no statistically significant association. Lastly, pressure sores developed in a median of 3 days, with a non-significant trend towards faster development in males and stage 1 ulcers, contrasting with a larger study’s significant stage-based differences.

This study showed that most ICU patients with pressure ulcers were older adults, although this difference was not statistically significant compared to those without pressure ulcers. This finding contradicts previous studies that reported older age as a risk factor for pressure sores in ICU patients [3,19,21]. These discrepancies in results may stem from the inclusion of younger patients in this study, variations in patient care procedures, cultural factors, and differences in study sizes. Older age independently correlated with the occurrence of pressure sores. The progressively rising proportion of very old residents represents a conspicuous influx of high-risk patients, characterized by the accumulation of chronic comorbidities, nutritional deficiencies, immobility, and aging skin [22,23]. However, this study’s documentation of comorbidities, dietary deficiencies, and immobility was incomplete. Males exhibited a statistically significant hazard ratio for pressure sore development, four times higher than females. This result was similar to the previous study. However, this study showed that a 1 kg/m^2^ increase in BMI increased the risk of pressure sore development by 0.22 times; this result was in contrast to previous studies, which reported that lower or decreased body weight was significantly associated with pressure sore development [3,19].

These findings concerning the health condition of ICU patients indicated that the length of ICU stays exceeding 9 days, increases in BMI, systolic blood pressure, and albumin levels, as well as masculinity, were statistically significant risk factors for pressure sore development. This outcome aligns with prior studies that highlighted that an extended ICU stay, along with factors such as diabetes, hypertension, and cardiovascular disease, increased the likelihood of pressure sore development among ICU patients [24,25]. However, this study did not establish a direct association between underlying conditions such as diabetes mellitus (DM) and hypertension (HT) and the development of pressure ulcers, as all patients with DM and HT developed pressure sores. Nevertheless, after adjusting for confounding factors using multivariable Cox regression analysis, neither DM nor HT emerged as significant hazards influencing the time to pressure sore development.

The treatment of critical patients in the ICU, including mechanical ventilation, the use of vasopressors, and sedative drugs, has been identified as a potential risk factor for pressure sore development, as reported in previous studies [26,27]. Research on mechanical ventilation has highlighted that using a ventilator for 10 h significantly impacts the occurrence of pressure sores [27]. However, this study’s results indicated that all ICU patients were on ventilators and did not show a statistically significant association with pressure sore development. This discrepancy could stem from patient conditions or variations in patient care procedures.

The overall median survival time for the development of pressure sores was 3 days. Males exhibited a shorter survival time for the development of pressure sores than females, although this difference was not statistically significant. The Kaplan–Meier analysis revealed that the survival time for pressure sore development in stage 1 was shorter than in other stages; however, this difference did not reach statistical significance. This contrasts with a more extensive previous study, which reported statistically significant differences in survival time for pressure sores across different stages using a log-rank test [19]. These variations in results may be attributed to differences in patient care procedures, cultural factors, and variations in study sizes.

## 5. The Strengths and Limitations of the Study

This study has several limitations. First, the data represent only a single point in time [20] and do not account for potentially influential factors such as staffing levels. Second, the generalizability of the findings is limited, as the study was conducted in a single hospital in Northeastern Thailand, which may not represent other geographic areas. Third, assessing pressure sore staging is inherently challenging; reliance on medical records and assistant nurses’ documentation may introduce uncertainty in tissue assessments. Although efforts were made to ensure consistency through standardized data collection procedures and training, some variability and bias may have occurred. Lastly, the small sample size and single-center design limit the ability to generalize the findings and preclude causal inference.

The strength of this study lies in its identification of foundational risk factors associated with the development of pressure sores. The findings provide essential evidence to support initiatives to address this critical patient safety issue and offer baseline data relevant for local and regional quality improvement efforts. Importantly, pressure sore staging was assessed through direct skin inspection—the gold standard—by trained outcome assessors, ensuring the reliability of outcome classification. The study was conducted following a rigorous protocol, with meticulous attention to standardization of data collection procedures. These results contribute valuable insight into risk stratification among ICU patients and may inform the development of clinical practice guidelines. Further studies involving larger and more heterogeneous populations are warranted to explore potential causal relationships.

## 6. Conclusions

Overall, this study offers several notable benefits. It precisely identifies that pressure sores typically develop within a median of just 3 days for ICU patients. This finding provides vital intelligence, allowing medical staff to address and manage this complication preemptively. The finding that males face a heightened risk, evidenced by a gender-based disparity in pressure sore development time, provides critical guidance for tailoring prevention strategies. Furthermore, pinpointing the coccyx as the most common site for these sores helps healthcare providers concentrate their preventative actions more effectively. Finally, the finding that patients staying in the ICU for nine days or longer have a significantly higher likelihood of developing pressure sores informs the deployment of more focused and proactive care strategies.

## Figures and Tables

**Table 1:** Characteristics and health conditions of the participants.

Factors	Total (n = 50)		Pressure Sore (n = 29)		No Pressure Sore (n = 21)		Exact/ r*
	n	(%)	n	(%)	n	(%)	
**Gender**							
Male	38	(76.00)	20	(52.60)	18	(47.40)	0.201
Female	12	(24.00)	9	(75.00)	3	(25.00)	
**Age (Years)** Mean, (S.D.)	56.74	(11.53)	57.62	(14.95)	55.52	(3.22)	<0.001 *
≤50 years	11	(22.00)	10	(90.90)	1	(9.10)	0.012 *
>50 years	39	(78.00)	19	(48.70)	20	(51.30)	
**BMI (Kg/M^2^)** Mean (S.D.)	22.16	(4.53)	22.93	(5.52)	21.10	(2.37)	0.160
<23.0 Kg/M^2^	35	(70.00)	17	(48.60)	18	(51.40)	0.039*
≥23.0 Kg/M^2^	15	(30.00)	12	(80.00)	3	(20.00)	
**Temperature (°C)** Mean, (S.D.)	37.89	(0.94)	38.02	(0.99)	37.73	(0.86)	0.290
(<37.0) **°C**	14	(28.00)	8	(57.10)	6	(42.90)	0.939
(≥37.5) **°C**	36	(72.00)	21	(58.30)	15	(41.70)	
**Systolic blood pressure (mmHg)** Mean, (S.D.)	126.00	(27.91)	127.65	(25.08)	123.71	(31.91)	0.627
≤140 mmHg.	34	(68.00)	22	(64.70)	12	(35.30)	0.161
>140 mmHg.	16	(32.00)	7	(43.80)	9	(56.20)	
**Diastolic blood pressure (mmHg)**							
Mean, (S.D.)	72.70	(16.39)	72.48	(13.65)	73.00	(19.93)	0.914
≤90 mmHg.	44	(88.00)	26	(59.10)	18	(40.90)	0.672
>90 mmHg.	6	(12.00)	3	(50.00)	3	(50.00)	
**Edema**	NA	NA	NA	NA	NA	NA	
**Urinary incontinence**	NA	NA	NA	NA	NA	NA	
**MAP** Mean, (S.D.)	87.26	(16.10)	86.31	(10.68)	88.57	(21.75)	0.629
Abnormal (MAP < 65 mmHg)	1	(2.00)	1	(100.00)	0	(0.00)	NA
Normal (MAP = 65–110 mmHg)	49	(98.00)	28	(57.10)	21	(42.90)	
**Inotrope**							0.917
Yes	29	(58.00)	17	(58.60)	12	(41.40)	
No	21	(42.00)	12	(57.10)	9	(42.90)	
**Restraining**							0.960
Yes	43	(86.00)	25	(58.10)	18	(41.90)	
No	7	(14.00)	4	(57.10)	3	(42.90)	
**ADL**							NA
Yes	50	(100.00)	29	(58.00)	21	(42.00)	
No	0	(0.00)	0	(0.00)	0	(0.00)	
**Ventilator**							
Yes	50	(100.00)	40	(80.00)	10	(20.00)	NA
No	0	(0.00)	0	(0.00)	0	(0.00)	
**Care**							NA
Doctor	0	(0.00)	0	(0.00)	0	(0.00)	
Nurse	50	(100.00)	40	(80.00)	10	(20.00)	
Cousin	0	(0.00)	0	(0.00)	0	(0.00)	
Others	0	(0.00)	0	(0.00)	0	(0.00)	
**Sleep**							0.726
Yes	30	(60.00)	18	(60.00)	12	(40.00)	
No	20	(40.00)	11	(55.00)	9	(45.00)	
Smoke	0	(0.00)	0	(0.00)	0	(0.00)	
**Defecation (time per day)**							0.520
No	0	(0.00)	0	(0.00)	0	(0.00)	
1 time	38	(76.00)	23	(60.50)	15	(39.50)	
Over 1 time	12	(24.00)	6	(50.00)	6	(50.00)	
**Underlying disease**							
DM (Diabetes mellitus)	15	(30.00)	15	(100.00)	0	(0.00)	<0.001 *
No	35	(70.00)	14	(40.00)	21	(60.00)	
HT (Hypertension)	12	(24.00)	12	(100.00)	0	(0.00)	0.001 *
No	38	(76.00)	17	(44.70)	21	(55.30)	
ESRD (End-stage renal disease)	3	(6.00)	3	(100.00)	0	(0.00)	0.254
No	47	(94.00)	26	(55.30)	21	(44.70)	
DLP (Dyslipidemia)	2	(4.00)	2	(100.00)	0	(0.00)	0.503
No	48	(96.00)	27	(56.30)	21	(43.80)	
CKD (Chronic kidney disease)	1	(2.00)	1	(3.405)	0	(0.00)	1.000
No	49	(98.00)	28	(57.10)	21	(42.79)	
CVA (Cerebrovascular accident)	1	(2.00)	1	(3.405)	0	(0.00)	1.000
No	49	(98.00)	28	(57.10)	21	(42.79)	
AAA (Abdominal aortic aneurysm)	1	(2.00)	1	(6.25)	0	(0.00)	1.000
No	49	(98.00)	28	(57.10)	21	42.90	
TB (Tuberculosis)	3	(6.00)	3	(100.00)	0	(0.00)	0.254
No	47	(94.00)	26	(55.30)	21	(44.70)	
Cancer	49	(98.00)	1	(3.405)	0	(0.00)	1.000
No	1	(2.00)	28	(57.10)	21	(42.79)	
**Comorbidity**	NA	NA	NA	NA	NA	NA	
**LOC**	NA	NA	NA	NA	NA	NA	
**Degree of pressure sore (Barden score)**							
Stage 1	11	(22.00)	11	(100.00)	0	(0.00)	NA
Stage 2	14	(28.00)	14	(100.00)	0	(0.00)	
Stage 3	4	(8.00)	4	(100.00)	0	(0.00)	
No	21	(42.00)	0	(0.00)	0	(0.00)	
**Time to event (day)** mean, (S.D.)	3.66	(4.90)	3.66	(4.90)	0	(0.00)	0.002 *
**Length of stay (day)** Mean, (S.D.)	7.60	(8.03)	10.21	(9.51)	4.00	(2.79)	0.006 *
≤9 Days	37	(74.00)	16	(43.20)	21	(56.80)	<0.001 *
>9 Days	13	(26.00)	13	(100.00)	0	(0.00)	
**Albumin** Mean, (S.D.)	2.61	(0.71)	2.67	(0.78)	2.53	(0.63)	0.491
Normal (3.5–5 gm/dL)	4	(8.00)	4	(100.00)	0	(0.00)	0.129
Abnormal (<3.5 gm/dL or >5 gm/dL)	46	(92.00)	25	(54.20)	21	(45.70)	
**Hemoglobin** Mean, (S.D.)	10.36	(1.99)	9.03	(1.36)	12.20	(1.04)	<0.001
Normal (>11.0 g/dL)	21	(42.00)	0	(0.00)	21	(100.00)	<0.001 *
Abnormal (<11.0 g/dL)	29	(58.00)	29	(100.00)	0	(0.00)	

*r = Pearson correlation coefficiency, BMI = Body Mass Index, MAP = mean arterial pressure, LOC = level of consciousness, ADL = activities of daily living, NA = Not available, * Significant level as *p*-value ≤ 0.05

**Table 2:** Survival time comparison between males and females.

Means and Medians for Survival Time								
Gender	Mean				Median			
	Estimate	Std. Error	95% Confidence Interval		Estimate	Std. Error	95% Confidence Interval	
			Lower Bound	Upper Bound			Lower Bound	Upper Bound
Male	4.20	0.76	2.72	5.68	3.000	0.36	2.28	3.72
Female	4.56	2.16	1.32	9.79	5.000	0.42	4.18	5.82
Overall	4.62	0.83	2.99	6.26	3.000	0.49	2.04	3.96

Estimation is limited to the largest survival time if it is censored.

**Table 3:** Hazard factors of pressure sores among ICU patients by multivariable Cox proportional regression.

Factor	B	SE	Wald	df	*p*-Value	HR	95.0% CI for HR	
							Lower	Upper
Male	1.359	0.579	5.508	1	0.019 *	3.891	1.25	12.10
BMI	0.215	0.064	11.441	1	0.001 *	1.240	1.09	1.40
Systolic	−0.035	0.012	8.567	1	0.003 *	0.966	0.94	0.99
DM	−0.954	0.554	2.963	1	0.085	0.385	0.13	1.14
Albumin	1.148	0.424	7.332	1	0.007 *	3.151	1.37	7.23
Length of stay	2.112	0.627	11.337	1	0.001 *	8.269	2.42	28.28

SE = standard error, HR = hazard ratio, * *p*-value ≤ 0.05.

## Data Availability

Data are available upon request.
